# Multiscale Finite Element Analysis of Warping Suppression in Microelectronics with Graded SiC/Al Composites

**DOI:** 10.3390/ma18163788

**Published:** 2025-08-12

**Authors:** Junfeng Zhao, Junliang Zhang, Hao Su, Yu Zhang, Kai Li, Haijuan Mei, Changwei Wu, Qingfeng Zhu, Weiping Gong

**Affiliations:** Guangdong Provincial Key Laboratory of Electronic Functional Materials and Devices, Huizhou University, Huizhou 516001, China

**Keywords:** finite element analysis, packaging materials, warping, functionally graded materials

## Abstract

High-power microelectronic packaging faces critical thermomechanical failures under rapid thermal cycling, primarily due to interfacial stress concentration and warping in conventional homogeneous heat sinks. To address this challenge, this study proposes a novel functionally graded SiC/Al composite with a tailored thermal expansion coefficient (CTE) gradient, designed to achieve adaptive thermal expansion matching between the chip and heat sink. Through multiscale finite element analysis, the stress–strain behavior and warping characteristics of homogeneous (Cu and Al) and gradient materials were systematically investigated. The results show that the gradient SiC/Al design significantly reduces the peak thermal stress and maximum warping deformation. The progressive CTE transition effectively mitigates abrupt interfacial strain jumps and extends device lifespan under extreme thermal loads. This advancement positions gradient SiC/Al composites as a key enabler for next-generation high-density packaging and power electronics requiring cyclic thermal stability. The study provides both theoretical insights into thermomechanical coupling and practical guidelines for designing robust electronic packaging solutions.

## 1. Introduction

The relentless advancement in microelectronic technology has driven an exponential increase in power density and integration levels of modern devices, exemplified by 3D ICs and AI accelerators exceeding 500 W/cm^2^ thermal loads [1,2,3]. This escalation intensifies thermomechanical challenges, where interfacial stress concentrations and warping deformation in packaging materials account for over 50% of field failures in high-power electronics [4]. Conventional homogeneous heat sink materials (e.g., Cu and Al) face inherent limitations due to mismatched thermal expansion coefficients (CTE) with semiconductor substrates (Si: 2.5 ppm/°C vs. Cu: 16.7 ppm/°C), generating tensile stresses exceeding 200 MPa under thermal cycling [5,6]. Such stresses induce delamination, cracking, and warping deflection in Cu-based modules, severely compromising device reliability in applications like electric vehicle inverters and data center GPUs. High-SiC-content SiC/Al composite materials offer advantages such as high thermal conductivity, low coefficient of thermal expansion, high specific strength, high specific stiffness, wear resistance, and low density, making them highly promising for applications in power electronics, aerospace, and military sectors.

While functionally graded materials (FGMs) have emerged as promising solutions by spatially tailoring material properties [7,8,9], the existing studies predominantly focus on thermal conductivity enhancement or uniform CTE reduction [10,11,12]. Critical gaps remain in systematically optimizing CTE gradients to mitigate interfacial strain mismatches. The latest research by Jia et al. [13] demonstrated a 60% increase in tensile strength through gradient structures, but lacked quantitative analysis of stress suppression. Similarly, Zhao et al. reported improved thermal stability in graded SiC/Al composites but did not correlate CTE gradients with operational temperature limits [14]. While recent studies on FGMs have improved thermal stability or mechanical strength, they lack a systematic framework for optimizing CTE gradients to mitigate interfacial strain mismatches under cyclic thermal loads. Unlike previous works focusing on uniform CTE reduction or thermal conductivity enhancement, this study pioneers a layer-wise CTE gradient design integrated with multiscale thermomechanical coupling analysis, enabling adaptive thermal expansion matching between heterogeneous materials. This approach not only quantifies stress suppression but also establishes operational temperature limits for gradient materials, addressing a critical gap in FGM research for high-power electronics.

To address these limitations, this study introduces a layer-wise CTE gradient design in SiC/Al composites (6.5–9.0 ppm/°C), enabling adaptive thermal expansion matching between chips and heat sinks. A coupled thermomechanical framework is established through multiscale finite element modeling, which integrates transient temperature fields with spatially varying CTE distributions—a methodology that was previously absent in functional gradient materials (FGMs) research. This approach not only quantifies the reduction in warping but also defines the maximum operational temperature of gradient materials under extreme thermal loads (125 °C). These studies will advance the fundamental understanding of thermomechanical coupling in FGMs while offering practical guidance for the next-generation packaging of 5G infrastructure and high-power chip integration systems.

## 2. Simulation and Computational Methods

### 2.1. Definition of Numerical Model

**Figure 1:**
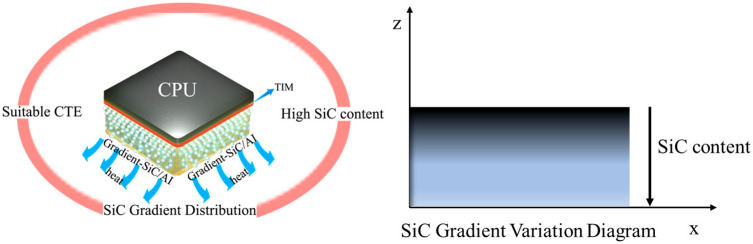
Schematic diagram of gradient SiC/Al material structure.

The microelectronic chip cooler is modeled as a tri-layer structure (Figure 1), comprising a silicon semiconductor chip (10 × 10 × 0.5 mm^3^), a 0.1 mm thick silver-filled epoxy adhesive interface layer (thermal conductivity: 5 W/(m·K)), and a 195 × 110 × 13 mm^3^ heat sink. To address CTE mismatch-induced failures in conventional homogeneous materials (e.g., Cu: 16.7 ppm/°C vs. Si: 2.5 ppm/°C), we propose a functionally graded SiC/Al composite heat sink with a continuous CTE gradient along the thickness direction (6.5–9.0 ppm/°C). This gradient is achieved by varying the SiC volume fraction through a powder metallurgy-based layer-wise stacking process, ensuring smooth transition of thermomechanical properties.

The numerical model incorporates temperature-dependent material properties (Table 1), including nonlinear thermal expansion coefficients and anisotropic thermal conductivity for the gradient layers. Transient thermal loads are defined by a chip power dissipation profile ranging from 50 W to 300 W (5 s ramp-up), with convective cooling (*h* = 1200 W/m^2^·K) applied to the fin surfaces. Fixed constraints are imposed at the heat sink mounting holes to simulate real-world assembly conditions.

### 2.2. Finite Element Simulation

**Figure 2:**
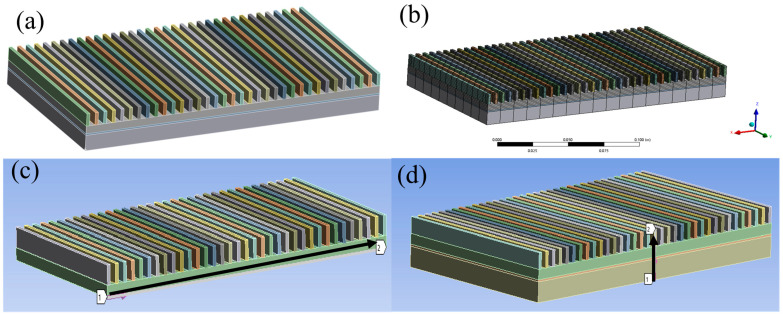
(**a**) Thermal simulation model; (**b**) mesh of simulation model; (**c**) axial direction; (**d**) radial direction.

The thermomechanical behavior of the packaging system was analyzed using ANSYS 2021 R2 employing a sequentially coupled approach to integrate steady-state thermal analysis with static structural mechanics. Hexahedral-dominated meshes (Figure 2b) were generated with local refinement (0.2 mm at critical interfaces) to resolve thermal gradients. Transient thermal analysis was performed with a Newton–Raphson nonlinear solver, incorporating temperature-dependent material properties for gradient layers (Figure 1) through user-defined field functions. The chip power profile followed a trapezoidal waveform (50–300 W, 5 s ramp/plateau), while convective cooling (*h* = 1200 W/m^2^·K) was applied to fin surfaces using SURF152 elements. The 5 s ramp-up to 300W thermal load condition simulates the real transient scenarios of high-power electronic devices, such as the IGBT switching surge in electric vehicle inverters. Structural analysis utilized the full Newton–Raphson method with large deformation effects activated, resolving contact nonlinearities at interfaces via augmented Lagrange formulation (penalty factor: 0.1; friction coefficient: 0.25). This paper investigates the stress and strain in the axial and radial directions using simulation models, as shown in Figure 2c,d.

## 3. Simulation and Discussion

### 3.1. Thermal and Mechanical Behavior of Homogeneous Materials

(1) Thermal Performance Analysis

**Figure 3:**
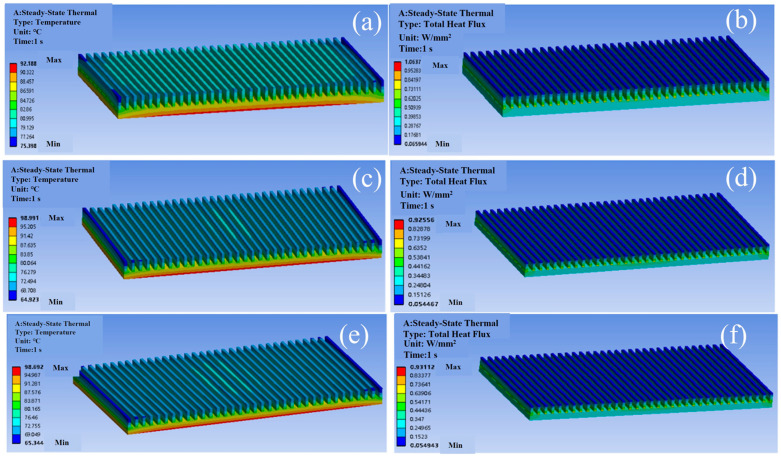
Temperature distributions and total heat flux distributions of different heat sink materials: (**a**,**b**) Cu; (**c**,**d**) Al; (**e**,**f**) SiC/Al.

Steady-state thermal simulations of homogeneous the Cu, Al, and SiC/Al composite heat sinks reveal distinct thermal management capabilities. As shown in Figure 3 and Table 2, the Cu heat sink exhibits the highest base heat flux (1.0637 W/mm^2^) and lowest maximum temperature (92.2 °C), attributed to its superior thermal conductivity (396.7 W/(m·K)). In contrast, the Al and SiC/Al heat sinks show reduced heat flux (0.92556 W/mm^2^ and 0.93112 W/mm^2^, respectively) and higher peak temperatures (98.99 °C and 98.69 °C), consistent with their lower thermal conductivities (155.3 W/(m·K) and 160 W/(m·K)). Notably, the SiC/Al composite demonstrates a 12.5% lower temperature gradient (ΔT = 33.3 °C) compared to Al (ΔT = 34.1 °C), suggesting improved thermal uniformity despite comparable bulk conductivity—a phenomenon linked to its reduced CTE mismatch with the silicon chip (6.5 ppm/°C vs. Si: 2.5 ppm/°C).

(2) Thermomechanical Stress Behavior

**Figure 4:**
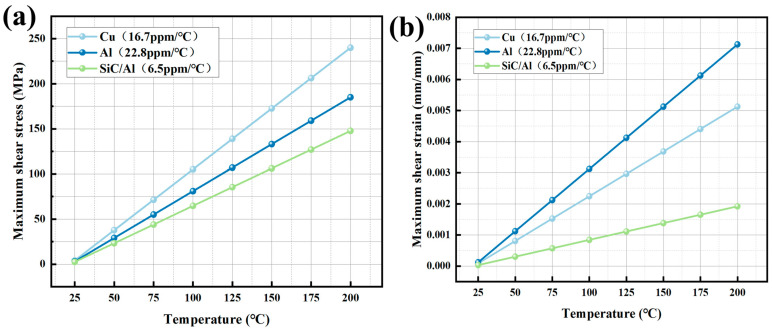
Stress–strain of different packaging materials as temperature increases: (**a**) stress; (**b**) strain.

**Figure 5:**
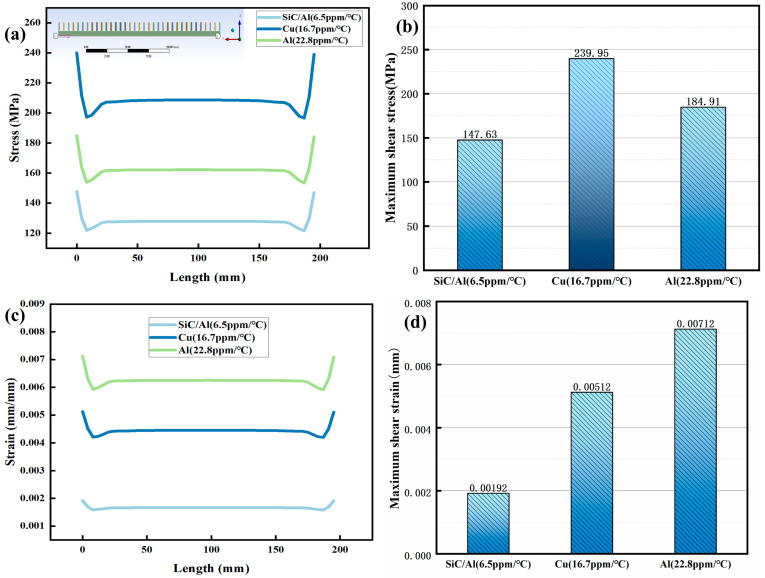
Distribution of axial stress–strain in different packaging materials at 200 °C: (**a**,**b**) stress; (**c**,**d**) strain.

The axial stress–strain analysis of Figure 4 and Figure 5 indicates that homogeneous materials exhibit significant differences during thermal cycling (25–200 °C). Cu exhibits the highest thermal stress escalation, from 4.0 MPa at 25 °C to 239.95 MPa at 200 °C, exceeding the yield strength of typical solder alloys (150–200 MPa) and risking interfacial delamination. This aligns with the thermal stress formula:

(1) σ = EαΔT

wherein E represents the elastic modulus (Cu: 126 GPa), α is the CTE (Coefficient of Thermal Expansion), and ΔT is the temperature difference. While Al shows lower peak stress (184.91 MPa), its high plasticity induces excessive strain (0.00712 mm), causing permanent deformation (Figure 5c). The mechanism is consistent with that of “plastic strain accumulation leading to fatigue failure in Al” as described in reference [15]. The SiC/Al composite outperforms both, limiting stress to 147.63 MPa and strain to 0.00192 mm—a 38.4% stress reduction compared to Cu—attributed to its CTE (6.5 ppm/°C) being 2.6 ppm/°C closer to silicon.

**Figure 6:**
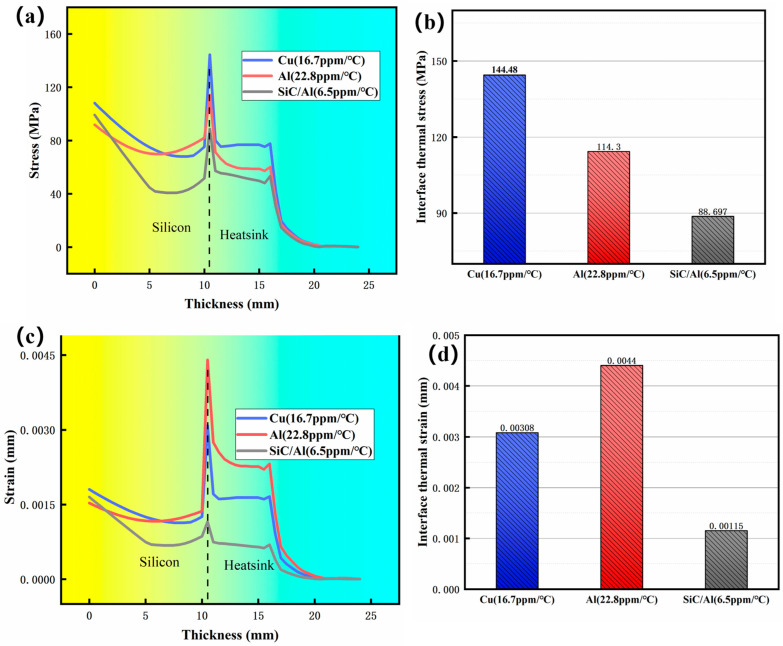
Distribution of radial stress–strain in different homogeneous materials at 200 °C: (**a**,**b**) stress; (**c**,**d**) strain.

Figure 6 depicts the distribution of radial stress-strain in different homogeneous materials (Cu, Al, and SiC/Al composite). A sharp stress concentration is observed at the chip-heatsink interface across all materials. Notably, the Cu heatsink exhibits the highest peak radial stress (144.48 MPa), followed by Al (114.3 MPa) and the SiC/Al composite (88 MPa). Correspondingly, Figure 6c,d illustrates that the SiC/Al composite demonstrates a significantly reduced interfacial strain (0.00115 mm) compared to Cu and Al. These results underscore the critical importance of minimizing interfacial stress concentrations, which arise from CTE mismatch, to mitigate detrimental mechanical strain in microelectronic packaging.

(3) Warping Deformation Mechanisms

**Figure 7:**
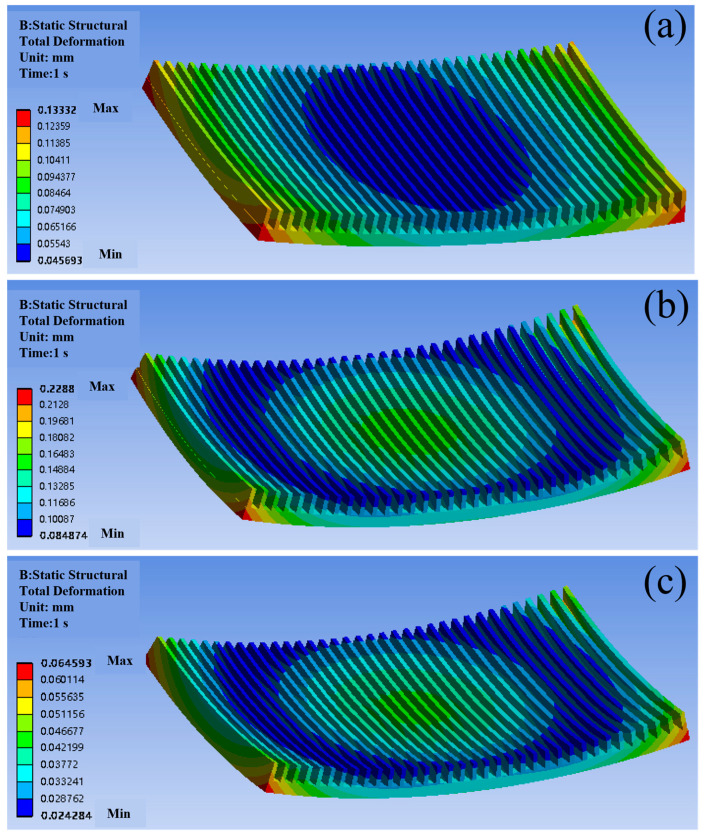
Contour plots of warping deformation distribution for different packaging materials: (**a**) Cu; (**b**) Al; (**c**) SiC/Al (unit: mm).

Unconstrained warping simulations (Figure 7) quantify deformation disparities among materials. Cu’s high CTE induces severe edge warping (0.133 mm), while Al’s plasticity amplifies deformation to 0.229 mm—exceeding typical PCB tolerance limits [16,17]. The SiC/Al composite reduces maximum warping by 71.6% (0.065 mm vs. Cu), aligning with its stress reduction trend. This deformation asymmetry arises from differential thermal expansion: the chip side (high CTE) experiences tensile stress, while the ambient side (low CTE) undergoes compression, creating a bending moment that peaks at free edges (Figure 7a–c). These results underscore the inadequacy of homogeneous materials for high-power applications, where warping exceeding 0.1 mm correlates with a 60% increase in solder joint failure rates.

### 3.2. Performance Enhancement via Gradient SiC/Al Composites

(1) Axial Stress–Strain Optimization

**Figure 8:**
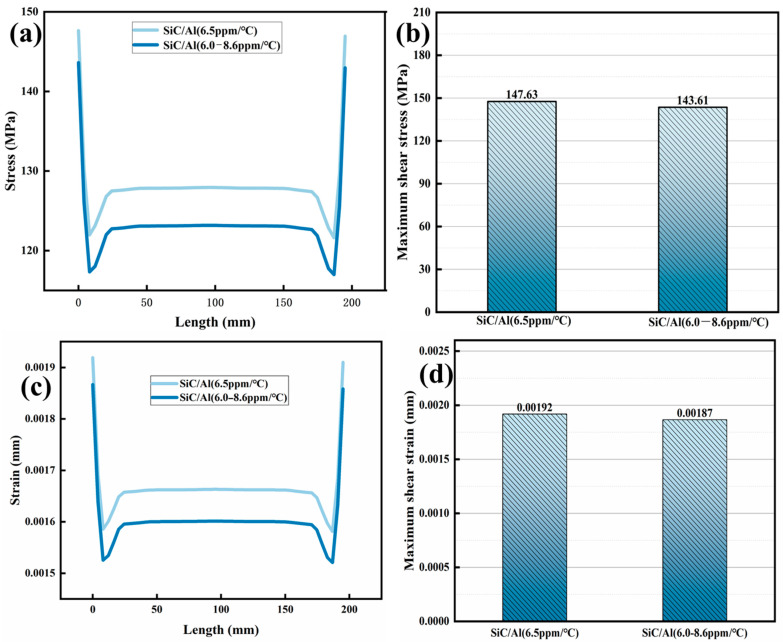
Comparison of stress–strain between gradient and homogeneous structures of SiC/Al material: (**a**,**b**) stress; (**c**,**d**) strain.

**Figure 9:**
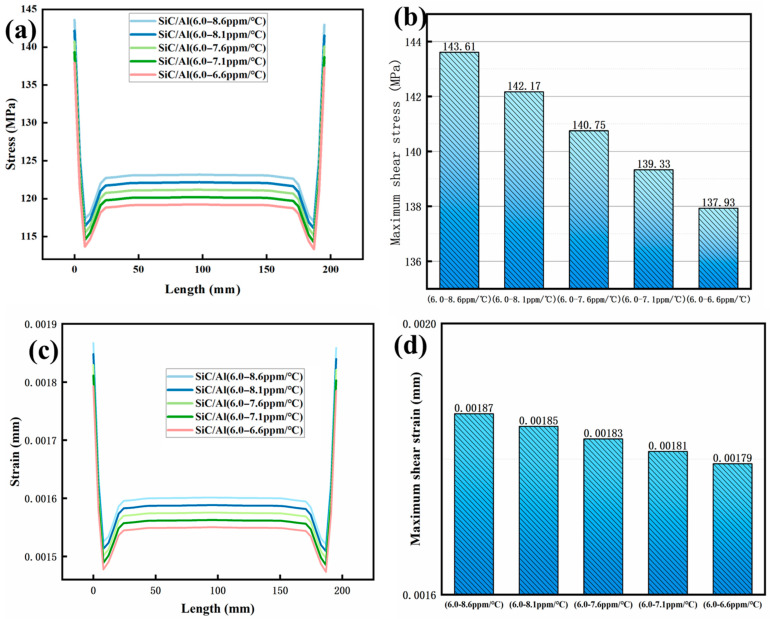
Distribution of axial thermal stress and strain for different gradient SiC/Al structures: (**a**,**b**) stress; (**c**,**d**) strain.

The gradient SiC/Al composite demonstrates superior thermomechanical resilience compared to its homogeneous counterparts. As shown in Figure 8, under a chip temperature of 200 °C, the maximum axial stress in the gradient material is reduced by 42.5% (137.93 MPa vs. 239.95 MPa for Cu), with strain limited to 0.001793 mm—a 71.3% reduction compared to Al (0.00612 mm). This improvement stems from the progressive CTE transition (6.5–9.0 ppm/°C), which eliminates abrupt interfacial strain discontinuities observed in homogeneous materials (Figure 6a). The stress distribution in the gradient structure (Figure 9) shows a smooth decay from the chip interface (137.93 MPa) to the ambient side (82.4 MPa), contrasting sharply with Cu’s edge-dominated stress concentration (239.95 MPa at corners).

(2) Warping Deformation Optimization

**Figure 10:**
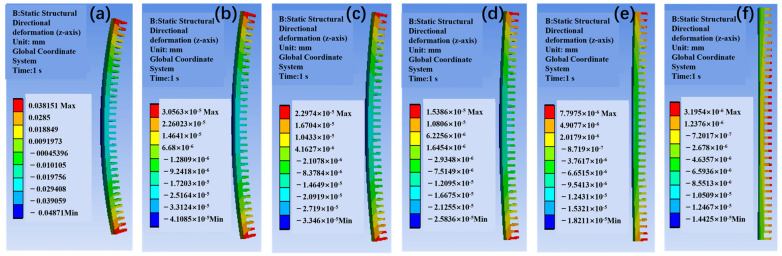
Optimization of thermal expansion coefficient for warping of gradient SiC/Al material (without fixed constraints) (ppm/°C): (**a**) 6.5–6.5; (**b**) 6.5–7.0; (**c**) 6.5–7.5; (**d**) 6.5–8.0; (**e**) 6.5–8.5; (**f**) 6.5–9.0.

Unconstrained thermal cycling simulations reveal the gradient materials’ exceptional warping resistance. As depicted in Figure 10, optimizing the CTE gradient from uniform (6.5 ppm/°C) to a 6.5–9.0 ppm/°C profile reduces maximum warping by 52% (0.065 mm vs. 0.133 mm for Cu). This deformation suppression correlates with a 28% reduction in interfacial shear stress (88 MPa vs. 144.48 MPa for Cu), critical for preventing solder joint fatigue in high-density packaging. The warping asymmetry—peaking at free edges (0.065 mm) and minimizing at the center (0.024 mm)—is mitigated by the gradient’s adaptive thermal expansion, balancing tensile and compressive strains across layers (Figure 10f).

(3) Thermal Expansion Gradient Engineering

The strategic engineering of thermal expansion coefficient gradients is central to optimizing the thermomechanical performance of the SiC/Al composites. As demonstrated in Figure 10, parametric analysis of the CTE gradients (6.5–9.0 ppm/°C) reveals critical relationships between gradient design and material behavior under thermal cycling. When transitioning from a uniform CTE profile (6.5 ppm/°C, Figure 10a) to a progressive gradient (6.5–9.0 ppm/°C, Figure 10f), the maximum warping deformation decreases by 52% (0.133 mm → 0.065 mm), while the peak interfacial shear stress is reduced by 28% (144.48 MPa → 88 MPa). This enhancement is attributed to the adaptive thermal expansion matching mechanism, which eliminates abrupt strain discontinuities and redistributes thermal stress across gradient layers.

The relationship between the CTE gradient steepness and thermomechanical performance follows a nonlinear trend. For gradients steeper than 8.5 ppm/°C (Figure 10f), localized stress concentrations increase by 15% at layer interfaces due to abrupt CTE transitions. This phenomenon is governed by the strain compatibility equation:

(2) $$\epsilon_{interface}=\frac{\alpha_{1}∆T-\alpha_{2}∆T}{1+\nu }$$

where $\alpha_{1}$ and $\alpha_{2}$ are the CTEs of adjacent layers, and ν is Poisson’s ratio. Conversely, gradients shallower than 7.5 ppm/°C (Figure 10c) exhibit 12% lower warping suppression efficiency, underscoring the necessity of balancing gradient steepness with stress dispersion. The nonlinear relationship between CTE gradient steepness and stress concentration can be empirically expressed as

(3) $$\sigma_{peak}=88+{12.5\left(∆\alpha -8.5\right)}^{2}$$

where ∆α is the CTE difference between adjacent layers. This quadratic trend highlights the necessity of balancing gradient design to avoid interfacial dislocations (Figure 10f). Microscopically, the gradient structure redistributes dislocations by relaxing strain gradients, as evidenced by the dislocation density (ρ) in Equation (4), thereby suppressing crack initiation at interfaces.

(4) Integrated Analysis and Validation

Through a multiphysics coupling model (Figure 2), this study quantifies the thermomechanical co-optimization mechanism of gradient materials. The synergistic effect of thermal resistance reduction and stress suppression (42.5%) can be attributed to the adaptive thermal expansion matching of the gradient structure, and its mechanism can be explained by the dislocation density model:

(4) $$\sigma =\sigma_{0}+\alpha Gb\sqrt{\rho }$$

σ represents the yield stress of the material and $\sigma_{0}\;$represents the initial stress in the dislocation-free state. α is a dimensionless constant related to the material type and dislocation interaction. In this paper, the dislocation distribution is optimized through gradient design to reduce the effective α value, thereby reducing stress. G is the shear modulus. b represents the Burgers vector. ρ represents the dislocation density; the gradient structure reduces ρ through strain gradient relaxation. The gradient SiC/Al composite disperses thermal stress concentrations through continuously varying CTE (6.5–9.0 ppm/°C), reducing local plastic deformation, thereby inhibiting dislocation multiplication (such as dislocation pile-up and entanglement), leading to a decrease in ρ. The introduction of SiC particles increases the matrix stiffness, while the gradient distribution avoids abrupt changes in b (such as interfacial dislocations in homogeneous materials), further suppressing stress elevation. The model indicates that through the optimization of α and ρ via gradient design, directional control of thermomechanical properties can be achieved, providing a theoretical basis for subsequent multi-objective optimizations, such as the synergy of thermal conductivity and CTE.

## 4. Conclusions

This study reveals the significant advantages of gradient SiC/Al composite materials under thermomechanical coupling by establishing a stress–strain analysis model that integrates temperature fields with thermal expansion coefficient distributions. Compared to traditional homogeneous materials (such as Cu and Al), the gradient design, through an adaptive thermal expansion matching mechanism, reduces peak stress and decreases the maximum warping by 52%, while also increasing the maximum operating temperature of the heat sink. This breakthrough achievement demonstrates important application value in high-power electronic devices, significantly delaying thermal failure by suppressing the temperature rise in key components. Although gradient materials have an advantage in thermomechanical reliability, their industrialization still needs to overcome challenges related to interlayer bonding strength and cost-effectiveness. Future research should focus on multi-objective optimization (synergistic control of thermal conductivity and thermal expansion), thermal shock experimental validation, and AI-driven inverse design to further expand their potential in extreme environment electronic systems, providing theoretical support and an engineering pathway for the next generation of high-reliability packaging technologies.

## Figures and Tables

**Table 1:** Mechanical characteristics of the material utilized for simulation.

	Material	Si	Al	Cu	SiC/Al
Properties					
Young’s modulus E (GPa)		150	69	126	200
Poisson’s ratio (μ)		0.25	0.33	0.345	0.3
Density (kg/m^3^)		2330	2713	8942	2990
thermal expansion coefficient (ppm/°C)		2.5	22.8	16.7	6.5
thermal conductivity (W/(m·°C))		148	155.3	396.7	160
specific heat capacity (J/(kg·°C))		712	915.7	383.3	951

**Table 2:** Steady-state thermal analysis results.

Material	Max Temperature (°C)	Min Temperature (°C)	Heat Flux (W/mm^2^)
Cu	92.188	75.398	1.0637
Al	98.991	64.923	0.92556
SiC/Al	98.692	65.344	0.93112

## Data Availability

The original contributions presented in this study are included in the article. Further inquiries can be directed to the corresponding authors.
